# Identification of Susceptibility Variants in *ADIPOR1* Gene Associated with Type 2 Diabetes, Coronary Artery Disease and the Comorbidity of Type 2 Diabetes and Coronary Artery Disease

**DOI:** 10.1371/journal.pone.0100339

**Published:** 2014-06-26

**Authors:** Zening Jin, Lianmei Pu, Liang Sun, Weijun Chen, Nan Nan, Hong Li, Huagang Zhu, Xinmiao Yang, Nana Wang, Juan Hui, Yurong Zhang, Qin Zhou, Fan Zhao, Fan Yang, Xiaohong Shi, Xiaoquan Zhu, Yige Yang, Wandong Zhang, Chenguang Zheng, Xiang Li, Duo Yang, Ruofei Jia, Shuai Meng, Ze Yang

**Affiliations:** 1 Department of Emergency Medicine, Anzhen Hospital, Capital Medical University, Beijing Institute of Heart Lung and Blood Vessels, Beijing, China; 2 The Key Laboratory of Geriatrics, Beijing Hospital & Beijing Institute of Geriatrics, Chinese Ministry of Health, Beijing, China; 3 Department of Cardiology, Beijing Ditan Hospital, Capital Medical University, Beijing, China; 4 Department of Cardiology, Beijing Anzhen Hospital, Capital Medical University, Beijing Institute of Heart Lung and Blood Vessels, Beijing, China; 5 Department of Functional Tests, Beijing You’an Hospital, Capital Medical University, Beijing, China; 6 Department of Neurology, Jiangbin hospital, Nanning, China; 7 Human Health Therapeutics, Institute for Biological Sciences, National Research Council of Canada, Ottawa, Canada; 8 Department of Cardiac Surgery, Guangxi Zhuang Autonomous Region Women and Children Care Hospital, Nanning, China; National Cancer Institute, National Institutes of Health, United States of America

## Abstract

**Objective:**

Adiponectin receptor 1 (encoded by *ADIPOR1*) is one of the major adiponectin receptors, and plays an important role in glucose and lipid metabolism. However, few studies have reported simultaneous associations between *ADIPOR1* variants and type 2 diabetes (T2D), coronary artery disease (CAD) and T2D with CAD. Based on the “common soil” hypothesis, we investigated whether *ADIPOR1* polymorphisms contributed to the etiology of T2D, CAD, or T2D with CAD in a Northern Han Chinese population.

**Methods:**

Our multi-disease comparison study enrolled 657 subjects, including 165 with T2D, 173 with CAD, 174 with both T2D and CAD (T2D+CAD), and 145 local healthy controls. Six *ADIPOR1* single nucleotide polymorphisms (SNPs) were genotyped and their association with disease risk was analyzed.

**Results:**

Multi-case-control comparison identified two *ADIPOR1* variants: rs3737884-G, which was simultaneously associated with an increased risk of T2D, CAD, and T2D+CAD (*P*-value range, 9.80×10^−5^−6.30×10^−4^; odds ratio (OR) range: 1.96–2.42) and 16850797-C, which was separately associated with T2D and T2D+CAD (*P*-value range: 0.007–0.014; OR range: 1.71–1.77). The risk genotypes of both rs3737884 and 16850797 were consistently associated with common metabolic phenotypes in all three diseases (*P*-value range: 4.81×10^−42^−0.001). We observed an increase in the genetic dose-dependent cumulative risk with increasing risk allele numbers in T2D, CAD and T2D+CAD (*P*_ trend_ from 1.35×10^−5^−0.002).

**Conclusions:**

Our results suggest that *ADIPOR1* risk polymorphisms are a strong candidate for the “common soil” hypothesis and could partially contribute to disease susceptibility to T2D, CAD, and T2D with CAD in the Northern Han Chinese population.

## Introduction

Coronary artery disease (CAD), type 2 diabetes (T2D), and T2D with CAD are multifactorial diseases in which hereditary and environmental factors both contribute to their etiology. These diseases may have a common pathogenesis based on the “common soil” hypothesis in which diabetes and cardiovascular disease share common antecedents [1]. Indeed, CAD, one of the main causes of death worldwide [2], and T2D together lead to the development of T2D with CAD.

Adiponectin receptor 1, encoded by the *ADIPOR1* gene, is a major adiponectin receptor that mediates the glucose and lipid metabolism-related effects of adiponectin on target cells. Research based on animal models has shown that *ADIPOR1* overexpression can augment the biological effects of adiponectin [3], whereas *ADIPOR1* knockout leads to increased insulin resistance (IR) and endogenous glucose production [4], suggesting a correlation between *ADIPOR1* expression and adiponectin activity [5]. Moreover, Wang *et al.* showed that down-regulated *ADIPOR1* signaling was the underlying mechanism for increased foam cell formation and accelerated cardiovascular disease in diabetic subjects [6].

Although several association studies reported that *ADIPOR1* variants were risk factors for IR [7], [8] or T2D [9]–[11], few studies investigated the relationship between *ADIPOR1* polymorphisms and CAD [12] or T2D with CAD [13]. In particular, there are limited reports about *ADIPOR1* variant simultaneous associations with T2D, CAD and T2D with CAD.

Based on the above-mentioned “common soil” hypothesis, we hypothesized that the etiology of T2D, CAD, and T2D with CAD could at least partially be associated with *ADIPOR1* polymorphisms, which may affect the interaction between receptor and ligand and thus play crucial roles in the development of genetic variants associated with these three diseases. We therefore conducted a multi-case-control association study to investigate the relationship between common *ADIPOR1* variants and the three diseases status in the Northern Han Chinese population.

## Materials and Methods

### Ethics Statement

This study complies with the Declaration of Helsinki, and the local ethics committees of the two participating hospitals (Beijing Anzhen Hospital, Capital Medical University, Beijing, China; Beijing Hospital & Beijing Institute of Geriatrics, Chinese Ministry of Health, Beijing, China) approved the research protocol. Written informed consent was obtained from each participant.

### Study Populations

This population-based, multi-case-control study was conducted on subjects who were permanent residents of the Beijing area, China, of self-identified Han ethnic origin. We enrolled a total of 657 individuals: 165 patients with T2D (T2D group), 173 with CAD (CAD group), 174 with both T2D and CAD (T2D+CAD group) and 145 healthy controls (Control group). Patients from the CAD and T2D+CAD groups were hospitalized at Beijing Anzhen Hospital between March 2007 and December 2009, while participants of the T2D and Control groups were recruited from Beijing Hospital between June and December 2008. Participant characteristics are given in Table 1.

**Table 1:** Clinical and demographic characteristics of participants among the four groups.

Characteristics	T2D+CAD (n = 174)	CAD(n = 173)	T2D(n = 165)	Control(n = 145)
Age (years)	62(55–69)	58(50–67)	62(51–69)	65(53–71)
Male, n(%)	121(69.5)	151(87.3)	121(73.3)	86(59.3)
BMI(kg/m^2^)	26.5±3.0	25.0±2.9	24.0±3.2	22.4±2.6
SBP (mmHg)	130(120–150)	120(119–130)	120(110–128)	110(104–120)
DBP(mmHg)	80(70–90)	77(70–80)	70(69–80)	70(60–76)
FPG(mmoL/L)	7.1(5.8–9.2)	5.3(4.9–5.7)	7.0(6.3–7.8)	5.1(4.9–5.3)
TG (mmoL/L)	1.6(1.1–2.3)	1.5(1.1–2.2)	1.2(1.0–1.6)	1.1(0.8–1.4)
TC (mmoL/L)	4.3±1.2	4.3±1.1	4.9±1.1	5.1±0.9
HDL-C (mmoL/L)	0.9(0.8–1.1)	0.9(0.8–1.1)	1.3(1.0–1.5)	1.6(1.4–1.9)
LDL-C (mmoL/L)	2.6(2.1–3.3)	2.7(2.1–3.5)	2.9(2.4–3.5)	2.7(2.3–3.3)

Variables are expressed as percentage, mean ± standard deviation, or median (interquartile range).

CAD, coronary artery disease; T2D, type 2 diabetes; T2D+CAD, T2D with CAD; BMI, body mass index; SBP, systolic blood pressure; DBP, diastolic blood pressure; FBG, fasting plasma glucose; TG, triglycerides; TC, total cholesterol; HDL, high-density lipoprotein cholesterol; LDL, low-density lipoprotein cholesterol.

T2D was diagnosed according to World Health Organization criteria [14], while classification of CAD patients was based on previous studies [15]. Patients of the T2D+CAD group met both the above-mentioned inclusion criteria. The inclusion criteria of the Control group were as follows: no ascertained diabetes, CAD, or T2D with CAD in first-degree relatives; no dyslipidemia, abnormal glucose tolerance, or high blood pressure; no potential CAD or myocardial infarction as determined from medical records or electrocardiographic tests. Subjects from all groups except the Control group underwent standard coronary angiography.

Individuals were excluded from the current study if: (1) they were ≤18 years old; (2) they had type 1 diabetes, diabetic ketoacidosis, hyperosmolar nonketotic diabetic coma, were relevantly autoantibody-positive, or needed insulin injections during the first year; (3) they had heart failure, myocardiopathy, or congenital heart disease; (4) they had autoimmune diseases (such as rheumatic diseases), cancer, infection, severe liver or renal diseases, were pregnant, or currently using glucocorticoid.

### Clinical Measurements and Biochemical Analyses

All study subjects were examined in the morning after an overnight fast to take anthropometric measurements and to collect blood samples for biochemical measurements and DNA extraction. Systolic blood pressure (SBP), diastolic blood pressure (DBP), height, weight, body mass index (BMI, calculated as weight in kilograms divided by the square of height in meters), plasma levels of total cholesterol (TC), triglycerides (TG), fasting plasma glucose (FBG), plasma high-density lipoprotein cholesterol (HDL-C) and plasma low-density lipoprotein cholesterol (LDL-C) were measured or calculated as previously described [16].

### Genotyping

Blood samples of participants were collected by standard venipuncture into evacuated vacuum tubes with ethylene diaminetetraacetic acid (EDTA). Genomic DNA was extracted from whole blood samples using standard DNA isolation methods [17]. The concentration and purity of the extracted DNA was determined by NanoDrop 2000c (Thermo Fisher, USA).

Single nucleotide polymorphisms (SNPs) in *ADIPOR1* were selected in the Han Chinese in Beijing (HCB) population provided on the International HapMap Project (HapMap Data PhaseIII/Rel#2, Feb09, on NCBI B36 assembly, dbSNP b126, http://hapmap.ncbi.nlm.nih.gov/). The selected region ranged from Chr1: g.202957430 (rs7514296) to Chr1: g. 202941190 (rs10581) (GRCh38), and the fragment length was 16240 bp. The gene borders were from the third intron to the 3′UTR. Tag SNPs were set to be minor allele frequency ≥0.05 and r^2^≥0.8 by Haploview V4.1. Tag SNPs were also not in linkage disequilibrium (LD) with each other (D’<0.8).

Six *ADIPOR1* tag SNPs were selected: rs7539542 (G>C), rs3737884 (C>T), rs1342387 (T>C), rs16850797 (G>C), rs12045862 (C>T) and rs7514221 (C>T). rs12045862 was genotyped by high resolution melting curves (HRM) analysis of PCR products while the remaining SNPs were genotyped using the PCR-restriction fragment length polymorphism (PCR-RFLP) method. Sequences of primer pairs, amplicon lengths, and annealing temperatures are listed in Table S1.

PCR was performed using a Bio-Rad C1000TM (Bio-Rad, USA). The 10 µL RCR reaction mixture for PCR-RFLP consisted of 0.1 µL forward and reverse primers (10 pmoL/µL), 1 µL DNA template, 1 µL 10×PCR Buffer, 0.1 µL Taq DNA polymerase (5 U/µL), 0.2 µL dNTP (10 mmoL/L), and deionized water to the total volume. PCR conditions were as follows: initial denaturation at 95°C for 5 min, followed by 35 cycles of denaturation at 95°C for 30 s, annealing for 30 s (Table S1), and extension at 72°C for 30 s, followed by 72°C for 7 min. Subsequently, PCR products were digested for 4 h at 37°C in a 10 µL mixture of 3 µL PCR product, 1 µL 10×PCR Buffer, 0.3 µL restriction enzyme (Table S1) and 5.7 µL deionized water, followed by detection on 8% ethidium bromide-stained polyacrylamide gel electrophoresis (Figure S1).

PCR reaction mixture for PCR-HRM was carried out in a total volume of 10 µL, including 0.18 µl 1×LC Green plus (Idaho, USA), 0.02 µl forward and 0.18 µL reverse primers (10 pmoL/µl), 1 µl DNA template, 1 µL 10×PCR Buffer, 0.1 µL Taq DNA polymerase (5 U/µL), 0.2 µL dNTP (10 mmoL/L), and deionized water to the total volume. PCR conditions were similar to those of PCR-RFLP but added two cycles at 95°C for 30 s, at 25°C for 2 min, at 94°C for 30 s, and at 24°C for 4 min. PCR products were transferred into HRM-specific 96-well plates, genotyped automatically, and verified manually using a LightScanner TMHR-I 96 (Idaho, USA) (Figure S1).

To check for errors in genotyping, 10% of each SNP samples were performed in duplicate and three samples of each genotype were randomly selected to be directly sequenced. The 50 µL reaction mixture included 0.5 µL of each primer (10 pmoL/µL), 5 µL DNA template, 5 µL 10×PCR Buffer, 0.5 µL Taq DNA polymerase (5 U/µL), 1 µL dNTP (10 mmoL/L), and deionized water to the total volume. PCR conditions involved initial denaturation at 95°C for 5 min, followed by 35 cycles at 95°C for 30 s, annealing for 30 s (Table S1), extension at 72°C for 45 s, followed by 72°C for 7 min. PCR products were subjected to electrophoresis on 8% polyacrylamide gels, visualized using the gel imaging system (Gel Doc2000, Bio-Rad, USA), and then sequenced by Beijing Tianyi Huiyuan Biosience & Technology Inc (Beijing, China). No discrepancies were observed. To minimize misclassification bias, genotyping was performed blindly to all other data.

### Statistical Analysis

**Figure 1:**
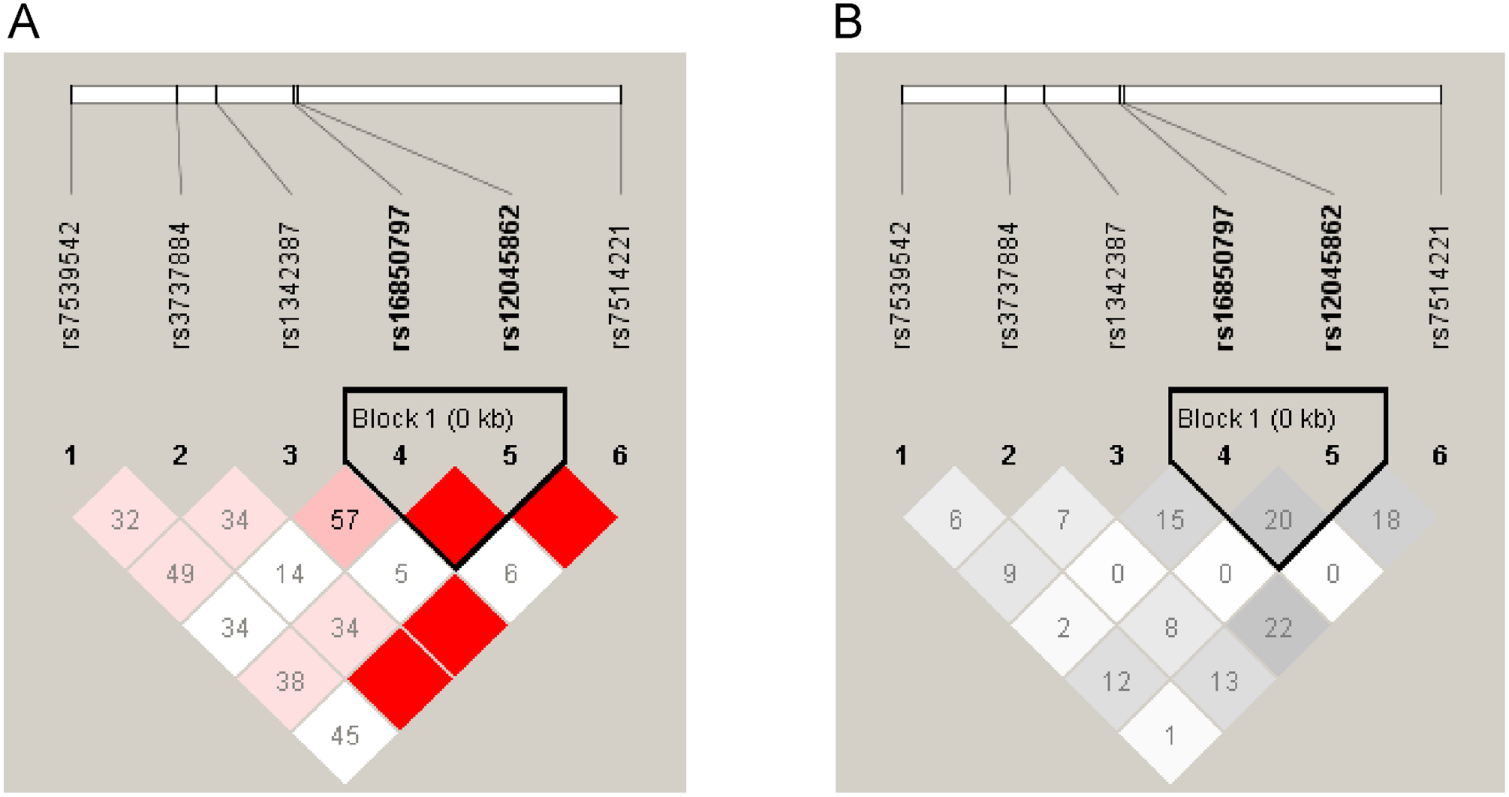
Linkage disequilibrium (LD) plots of 6 SNPs in *ADIPOR1* gene in Control group. A represents LD measure of D’; B represents LD measure of r^2^.

Continuous variables were presented as median values and interquartile ranges or means ± standard deviation, and categorical variables were presented as percentages, depending on the distribution of the variables. Differences in demographic and clinical characteristics between groups were compared using parametric (Student’s *t*-test or one-way ANOVA test for normally distributed variables) or nonparametric (Mann-Whitney U test or Kruskal-Wallis test for non-normally distributed variables) methods for continuous variables, and Pearson’s χ^2^ analysis or unconditional logistic regression for categorical variables. Individuals with missing data for a particular analysis were removed from the analysis. Tests for Hardy-Weinberg equilibrium (HWE) were performed separately for each SNP in control subjects using the online computer platform SHEsis (http://analysis.bio-x.cn/myAnalysis.php). Pairwise LD was performed by Haploview V 4.1 (Broad Institute, Cambridge, MA) (Figure 1). The risk-allele was designated 1 and the non-risk-allele 0. Genotypes were coded 0, 1 and 2, to represent the number of risk alleles carried by the subject, i.e., 2 represents carriers with two risk-alleles. Genotype frequencies were compared between cases and control subjects under the additive genetic model (comparing risk homozygous vs. heterozygous vs. wild homozygous carriers of variant) by unconditional logistic regression analysis adjusted for gender, age and BMI. The dominance genetic model (comparing risk homozygous + heterozygous vs. wild homozygous carriers of variant) and recessive genetic model (comparing risk homozygous vs. heterozygous wild + homozygous carriers of variant) analyses were also adopted. The cumulative effects of significant SNPs from single SNP analyses were calculated by counting the number of carriers with risk alleles. Odds ratios (OR) and 95% confidence intervals (CI) were used to estimate the strength of association between variables. The *P*-value for trend (*P*_trend_) was obtained by performing a χ^2^ test for linear trend in EpiInfo version 6. SPSS statistical software package version 19.0 was used for statistical analysis. *P*-values were based on a two-sided test with the significance level set at *P*≤0.05. Correction for multiple testing was performed by Bonferroni correction (http://www.quantitativeskills.com/sisa/calculations/bonfer.htm). Under this method, three independent hypotheses about a set of data were tested with a significance level set at *P*≤0.017 (0.05/3).

## Results

### Baseline Data Characteristics and Genotyping

Table 1 shows the demographic and clinical characteristics of participants in the different groups, including median ages (58–65 years), the percentage of males (57.3–87.3%), and BMI means (22.4–26.5 kg/m^2^), etc. Most of these data items were statistically different (*P*<0.05). Details of between-group comparisons were shown in Table S2. All SNPs examined in this study had three types of genotypes. PCR-RFLP (Figure S1) and PCR-HRM (Figure S2) results were consistent with those of sequencing analysis (Figure S3). Allelic frequencies are summarized in Table S3, and minor allele frequencies ranged from 0.11–0.40. Overall, each SNP conformed to HWE (*P*>0.05) in healthy controls, similar to that reported for HCB and Southern Han Chinese (CHS) in HapMap or Ensembl databases, but different from those reported for Utah residents with Northern and Western European ancestries from the CEPH collection (CEU) (Table S4). The Haploview program revealed that these SNPs were in a weak pairwise LD with each other (Figure 1), so we included all of them in further analyses.

### SNP Association Analysis

We analyzed the genotypic distribution of the six *ADIPOR1* SNPs using an additive model, and adjusted all data according to age, gender, and BMI. Two SNPs were found to be of particular interest after adjusting for covariates and performing the Bonferroni correction for multiple testing (Table 2): rs3737884, G was consistently associated with an increased risk of T2D (*P* = 6.30×10^−4^, OR(95%CI) = 1.96(1.33–2.89)), CAD (*P* = 9.80×10^−5^, OR(95%CI) = 2.42(1.55–3.77)), and T2D+CAD (*P* = 2.48×10^−4^, OR (95%CI) = 2.42(1.51–3.89)); while rs16850797, C was positively associated with both T2D (*P* = 0.007, OR (95%CI) = 1.77(1.17–2.67)) and T2D+CAD (*P* = 0.014, OR (95%CI) = 1.71(1.11–2.62)) (Table 2), but not with CAD (*P>*0.017).

**Table 2:** Association of SNPs in *ADIPOR1* with T2D, CAD and T2D with CAD in additive genetic model.

SNPs	Risk allele	Additive model	T2D+CAD vs. Control		CAD vs. Control		T2D vs. Control	
			OR (95% CI)	*P*	OR (95% CI)	*P*	OR (95% CI)	*P*
rs7539542	G	GG vs. GC vs. CC	1.24(0.83–1.85)	0.295	1.38(0.94–2.05)	0.104	0.97(0.69–1.36)	0.838
rs3737884	G	GG vs. GA vs. AA	2.42(1.51–3.89)	2.48×10^−4^ *	2.42(1.55–3.77)	9.80×10^−5^ *	1.96(1.33–2.89)	6.30×10^−4^ *
rs1342387	A	AA vs. AG vs. GG	1.09(0.72–1.67)	0.679	1.12(0.76–1.65)	0.568	1.12(0.79–1.57)	0.529
rs16850797	C	CC vs. CG vs. GG	1.71(1.11–2.62)	0.014*	1.21(0.79–1.87)	0.380	1.77(1.17–2.67)	0.007*
rs12045862	T	TT vs. TC vs. CC	1.07(0.69–1.67)	0.760	1.11(0.75–1.63)	0.614	1.32(0.92–1.90)	0.130
rs7514221	C	CC vs. CT vs. TT	1.68(0.90–3.15)	0.105	1.98(1.11–3.52)	0.021	1.73(1.02–2.96)	0.043

SNPs, single nucleotide polymorphisms; CAD, coronary artery disease; T2D, type 2 diabetes; T2D+CAD, T2D with CAD; OR, odds ratios; CI, confidence interval. All OR and *P* values are obtained by unconditional logistic regression and adjusted for gender, age and body mass index The *df* of a per-allele OR value is 2 in the additive genetic model analysis. All variants with nominal significance (*P*≤0.05) are listed; the threshold for significance by Bonferroni correction is 0.05/3 = 0.017 (three independent hypotheses: T2D vs. Control, CAD vs. Control, T2D with CAD vs. Control).

*P value that can pass Bonferron correction (*P*≤0.017).

Genetic analysis for the association of these two *ADIPOR1* SNPs with T2D, CAD, and T2D+CAD using dominant and recessive models achieved similar results to those seen for the additive model (Table S5).

### Stratification Analysis of Associated SNPs by T2D or CAD Status

To assess whether there was an overlapping genetic effect of the SNPs on CAD or T2D risk, we performed a case-case comparative analysis stratified by T2D or CAD status (Table 3). Genotype frequencies of rs3737884 showed no significant differences between T2D and T2D+CAD groups or between CAD and T2D+CAD groups (*P*>0.05). Additionally, the genotype frequencies of rs16850797 did not differ significantly between T2D and T2D+CAD groups (*P*>0.05). However, the genotype frequencies of rs16850797 reached statistical significance in a comparison of CAD patients with and without T2D (*P*_adjusted_ = 0.024, OR (95%CI) = 1.49 (1.05–2.10)) (Table 3).

**Table 3:** Association of rs3737884 and rs16850797 with diseases stratified by CAD or T2D status.

	Additive model	Unadjusted		Adjusted	
Comparisons	MM vs. Mm vs. mm^a^	OR(95%CI)^b^	*P* b	OR (95%CI)^c^	*P* c
rs3737884					
T2D	98/62/5	1.00(reference)	-	1.00(reference)	-
T2D+CAD	111/57/6	1.14(0.78–1.67)	0.509	1.12(0.74–1.71)	0.587
CAD	114/53/6	1.00(reference)	-	1.00(reference)	-
T2D+CAD	111/57/6	0.93(0.64–1.37)	0.726	0.91(0.60–1.39)	0.655
rs16850797					
T2D	8/89/63	1.00(reference)	-	1.00(reference)	-
T2D+CAD	28/73/73	1.22(0.88–1.703)	0.234	1.30(0.90–1.89)	0.163
CAD	13/64/96	1.00(reference)	-	1.00(reference)	-
T2D+CAD	28/73/73	1.62(1.19–2.22)	0.003	1.49(1.05–2.10)	0.024

CAD, coronary artery disease; T2D, type 2 diabetes; T2D+CAD, T2D with CAD; OR, odds ratios; CI, confidence interval.

a M/m represent G/A for rs3737884, and C/G for rs16850797.

b unadjusted for gender, age and body mass index;

c adjusted for gender, age and body mass index. Statistical significances are considered as *P*≤0.05. ORs are computed using T2D or CAD as the reference group. All OR and *P* values are obtained by unconditional logistic regression.

### Stratification Analysis of Associated SNPs by Clinical Phenotypes

To further explore the possible effect of risk *ADIPOR1* genotypes on clinical phenotypes, we selected and divided the subjects who carried risk genotypes into disease (T2D, CAD, and T2D+CAD) and healthy control subgroups. We defined the risk genotypes according to the dominance genetic model analysis in Table S5 and analyzed the association between clinical phenotypes and risk *ADIPOR1* genotypes, including rs3737884 (GG+AG) and rs16850797 (CC+CG).

The multi-case-control comparison found significantly higher BMI, SBP, DBP, FBG and lower HDL-C levels in cases with rs3737884 (GG+AG) genotypes among T2D (*P*-values from 0.001 to 4.81×10^−42^), CAD (*P*-values from 0.002 to 6.65×10^−39^) and T2D+CAD (*P*-values from 3.4×10^−13^ to 9.78×10^−38^) groups than in the Control group. These same phenotypes were also more significant in patients with rs16850797-specific genotypes (CC+CG) in T2D (*P*-values from 0.02 to 1.57×10^−12^) and T2D+CAD (*P*-values from 4.63×10^−10^ to 4.94×10^−33^) groups compared with the Control group. (Table S6).

### Cumulative Effect of Associated SNPs

Because haploview analysis did not reveal rs3737884 and rs16850797 to be in LD (Figure 1), we adopted a cumulative effect analysis to evaluate the genetic dose effect of risk alleles on T2D, CAD and T2D+CAD to clarify locus-locus interaction between risk alleles. As shown in Table 4, we observed an increase of the genetic dose-dependent cumulative risk with increased risk allele numbers. Overall, carriers with more than three risk alleles had a 2–3 fold risk for T2D (*P* = 1.64×10^−4^, OR (95%CI) = 2.93(1.67–5.17)), CAD (*P* = 0.002, OR (95%CI) = 2.40(1.35–4.23)), or T2D+CAD (*P* = 1.14×10^−5^, OR (95%CI) = 3.38 (1.95–5.87)) compared with Control individuals. Finally, we calculated the genetic dose-dependent cumulative risk, by comprising those cases and controls who harbored two or more than three risk alleles with carriers of one or no risk alleles. Significant *P*_trend_ values were obtained for all three disease groups: T2D (*P*_trend_ = 1.79×10^−4^), CAD (*P*_trend = _0.002), and T2D+CAD (*P*_trend_ = 1.35×10^−5^). (Table 4).

**Table 4:** Cumulative effect analyses of rs3737884 and rs16850797 in *ADIPOR1* associated with risk of T2D, CAD and T2D with CAD.

Number of risk alleles	T2D (N = 160^a^), n(%)	Control (N = 145), n(%)	OR(95%CI)	*P*	*P*_ trend_
≤1	36(22.50)	65(44.83)	1.00(reference)	-	-
2	59(36.88)	40(27.59)	2.66(1.50–4.72)	6.09×10^−4^	-
≥3	65(40.63)	40(27.59)	2.93(1.67–5.17)	1.64×10^−4^	1.79×10^−4^
Number of risk alleles	CAD (N = 173), n(%)	Control (N = 145), n(%)	OR(95%CI)	*P*	*P*_trend_
≤1	38(21.97)	65(44.83)	1.00(reference)	-	-
2	79(45.67)	40(27.59)	3.38(1.95–5.87)	1.14×10^−5^	-
≥3	56(32.37)	40(27.59)	2.40(1.35–4.23)	0.002	0.002
Number of risk alleles	T2D+CAD (N = 174), n(%)	Control (N = 145), n(%)	OR(95%CI)	*P*	*P*_trend_
≤1	38(21.84)	65(44.83)	1.00(reference)	-	-
2	57(32.76)	40(27.59)	2.44(1.38–4.31)	0.002	-
≥3	79(45.40)	40(27.59)	3.38(1.95–5.87)	1.14×10^−5^	1.35×10^−5^

Risk alleles are rs3737884, G and rs16850797, C, respectively. CAD, coronary artery disease; T2D, type 2 diabetes; T2D+CAD, T2D with CAD; OR, odds ratios; CI, confidence interval. OR values are calculated using those carrying one or no risk alleles as the reference group. OR and *P* values are obtained by Pearson’s χ^2^; *P*_ trend_, *P*-value for trend is obtained by performing a χ^2^ test for linear trend in EpiInfo version 6; All OR, *P* and *P*_ trend_ values are unadjusted for gender, age and body mass index; Statistical significances are considered as *P*≤0.05. ^a^Numbers of patients with T2D vary because of missing genotype data.

## Discussion

Using a multi-case-control comparison in a northern Chinese population, we identified two variants in the *ADIPOR1* gene, which supported our hypothesis that the etiology of CAD, T2D, and T2D with CAD was partially associated with *ADIPOR1* polymorphisms. rs3737884, G was shown to be associated with an increased risk of all three diseases, while rs16850797, C was associated with T2D and T2D with CAD. To the best of our knowledge, neither polymorphism has previously been reported in association with diseases.

Interestingly, we also found that the risk genotypes of both rs3737884 and rs16850797 were consistently associated with five common metabolic phenotypes in T2D, CAD, and T2D with CAD. Thus, it is conceivable that the *ADIPOR1* risk variants are not only shared by all three diseases status, but also by different metabolic phenotypes. This would result in a simplified, more consistent association between *ADIPOR1* and the three diseases status. Therefore, we postulate that the *ADIPOR1* variants act on the development of other metabolic disturbances such as IR, which partially contributes to the etiology of T2D, CAD, and T2D with CAD by fulfilling the “common soil” hypothesis.

Previously, Soccio *et al*. showed that three SNPs (including rs7539542) out of six tag SNPs selected from the HapMap-CEU database were significantly associated with CAD susceptibility among individuals with T2D [13]. Based on their analysis, when using major allele homozygote as the reference between CAD-positive and-negative T2D subjects in our data, we found that the GC genotype of rs7539542 was possibly, but not convincingly associated with an increased risk of CAD (GC vs. CC, *P* = 0.043, OR (95%CI) = 1.20(1.02–3.87); GG vs. CC *P* = 0.617, OR (95%CI) = 1.19(0.60–2.37)). Although the risk allele in both cases was G, it was the major allele in our analysis and the minor allele in the study by Soccio *et al.* [13]. Based on HapMap and Ensembl databases, we found that the allelic distribution of rs7539542 in the subjects of our study was similar to that of HCB and CHS, but significantly different from CEU (Table S4). This suggests that the different genetic background of various ethnic populations might affect CAD susceptibility and could be more informative for rs7539542 in European than in Asian populations.

We also found that the frequencies of rs3737884, G did not differ significantly between any of the disease groups, and that the frequencies of rs16850797, C were not significantly different between T2D and T2D+CAD groups. This case-only analysis therefore showed that shared *ADIPOR1* variants were not associated with disease status, which could reflect the fact that they posed a similar risk to the development of T2D, CAD, and T2D with CAD. It also indicates that rs3737884, G and rs16850797, C overlap slightly in their contribution to the etiology of these diseases, which further supports the “common soil” hypothesis that diabetes and cardiovascular disease share common antecedents.

Phenotype analyses showed that the risk genotypes of rs3737884 (GG+AG) and rs16850797 (CC+CG) were consistently associated with common metabolic phenotypes which were considered to be risk factors for the three diseases (Table S6). These were higher BMI, SBP, DBP, FBG, and lower HDL-C levels, which showed significant differences in the disease groups compared with the Control group. Our findings could prove the previously identified relationship between adiponectin and metabolic traits [16], which was also observed in animal models [4], [18]. *ADIPOR1* knockout mice had significantly impaired glucose tolerance and significantly higher plasma insulin concentrations compared with wild-type [4]. By contrast, macrophage-specific *ADIPOR1* transgenic mice showed reductions in whole body weight, TC, TG, and free fatty acid, and improved glucose tolerance and insulin sensitivity [18]. Because these metabolic traits are considered traditional risk factors for CAD, and as individuals with T2D often show these metabolic abnormalities, this indicates that T2D and CAD may share partial genetic susceptibility, which could include *ADIPOR1* polymorphisms. However, further functional pathogenesis studies are needed to confirm this.

Although some metabolic phenotypes are common to T2D, CAD, and T2D with CAD, there are few reported genetic variants shared by the three diseases. The present study identified such variants in *ADIPOR1* and our genotype-phenotype study showed that these variants were also associated with common metabolic phenotypes in T2D, CAD, and T2D with CAD. Thus, it is possible that the shared variants have a more extensive pathophysiological impact on disease, common metabolic phenotypes, and functional disturbance in the human body. Moreover, we speculate that the “common soil” of these diseases is in fact the common metabolic phenotypes and functional disturbances that lead to the development of T2D, CAD, and T2D with CAD. The risk genotypes shared by all three diseases could therefore contribute to disease susceptibility.

The observed trend of increased dose-dependent cumulative genetic risk with increasing risk allele numbers indicates that the risk alleles might be a form of quantity trait loci (QTL) that contributes to the risk of disease occurrence. Because genetic predisposition to complex disease is thought to reflect the cumulative effect of variants, our results suggest that susceptibility to T2D, CAD, or T2D with CAD is dose-dependent with increasing numbers of risk alleles. This could be explained by speculating that a higher number of risk variants have a potentially synergistic effect on interference with the interaction or affinity between receptors and ligands, affecting events within target cells such as signal transduction. Repeating our analysis on a larger cohort would be useful to confirm the associations observed here in a relatively small sample size and help us to better understand the underlying pathogenesis of the three diseases.

In summary, we identified *ADIPOR1* SNPs rs3737884 and rs16850797 as **s**hared genetic variants that were consistently associated with T2D, CAD, and T2D with CAD in a northern Chinese population. Our data suggests that *ADIPOR1* polymorphisms have a QTL risk and cause pleiotropic effects that occur during the development of the three diseases. These findings could provide novel insights into the etiology of metabolic diseases as well as the development of genetic markers to identify these diseases in a clinical setting.

## Supporting Information

Figure S1 **Amplification and restriction fragment length polymorphism picture of rs7539542 in** ***ADIPOR1*** **.** A) Representative PCR amplification gel picture of rs7539542. Lanes 1–7: 542 bp amplified PCR product. Lane marker: 100 bp DNA ladder marker. B) RFLP picture of rs7539542 after restriction digestion with BsmAIagarose gel electrphoresis. Lane 1, 2, 5: Homozygous wild genotype GG (542 bp); Lane 4: Homozygous variant genotype CC (349 bp, 193 bp); Lanes 3, 6: Heterozygous genotype GC (542 bp, 349 bp, 193 bp); Lane marker: 100 bp DNA ladder marker.
(TIF)

Figure S2 **Genotyping results of rs12045862 using LightScanner TMHR-I 96.** Melting curves results show homozygous wild genotype CC (blue curves), homozygous variant genotype TT (gray curves) and Heterozygous genotype CT (red curves).
(TIF)

Figure S3 **Sequencing results of rs1342387.** The positions that the arrow pointed to are homozygous wild genotype GG (A), heterozygous genotype GA (B), and homozygous variant genotype AA (C), respectively.
(TIF)

Table S1 **Basic information for genotyping.** F: forward primer; R: reverse primer; P: probe; HRM: high-resolution melting; RFLP: restriction fragment length polymorphism. *rs12045862 was genotyped using unlabeled probe method of HRM. **R:A or G;Y:C or T;N:A,C,G or T.
(DOC)

Table S2 **The multiple comparison results of clinical and demographic characteristics among groups.** Variables were expressed as percentage, mean ± standard deviation, or median (interquartile range); Differences between characteristics were compared using parametric (Student’s *t*-test for normally distributed variables) or nonparametric (Mann-Whitney U test for non-normally distributed variables) methods for continuous variables, and Pearson’s χ^2^ analysis for categorical variables. CAD, coronary artery disease; T2D, type 2 diabetes; T2D+CAD: T2D with CAD; HDL, high-density lipoprotein cholesterol; LDL, low-density lipoprotein cholesterol; SBP, systolic blood pressure; DBP, diastolic blood pressure; FBG, fasting plasma glucose; TC, total cholesterol; TG, triglycerides; BMI, body mass index. Six independent hypotheses are tested with a significance level set at *P*≤0.008 (0.05/6) according to Bonferroni correction.
(DOC)

Table S3 **Allelic distribution of the 6 SNPs in** ***ADIPOR1*** **in our study.** SNPs: single nucleotide polymorphism; CAD, coronary artery disease; T2D, type 2 diabetes; T2D+CAD: T2D with CAD. *These alleles were defined on the basis of the alleles contrast in this study. **The former is ancestral allele. *** UTR-3: untranslated region.
(DOC)

Table S4 **Allelic distribution of SNPs in our study, HapMap and Ensembl database.** HapMap database is from International HapMap Project (http://hapmap.ncbi.nlm.nih.gov/). Ensembl database is from the 1000 Genomes data (http://asia.ensembl.org/index.html). HCB or CHB: Han Chinese in Beijing, China. CHS: Southern Han Chinese. JPT: Japanese in Tokyo, Japan. CEU: Utah residents with Northern and Western European ancestry from the CEPH collection. YRI: Yoruba in Ibadan, Nigeria (Sub-Saharan African). Major: major allele frequency. Minor: minor allele frequency. HWE: Hardy-Weinberg equilibrium. The character “−” represents “unavailable”. *Numbers of sample size. **P values are derived from comparing the Control group with other groups, respectively. Statistical significances are considered as *P*≤0.013 (0.05/4, compared Control with HapMap database) or 0.01 (0.05/5, compared Control with Ensembl database). *P* values are obtained by Pearson’s χ^2^ analysis.
(DOC)

Table S5 **Association of rs3737884 and rs16850797 with diseases in three types of genetic models.** CAD, coronary artery disease; T2D, type 2 diabetes; T2D+CAD: T2D with CAD; OR, odds ratios; CI, confidence interval. All OR and *P* values are obtained by Pearson’s χ^2^ or unconditional logistic regression and adjusted for gender, age and body mass index. All variants with nominal significance (*P*≤0.05) are listed; the threshold for significance by Bonferroni correction is 0.05/3 = 0.017 (three independent hypotheses: T2D vs. Control, CAD vs. Control, T2D with CAD vs. Control).*P value that can pass multiple testing correction (*P*≤0.017); E indicates the power of the base-10 exponent (i.e. 9.80E-05 = 9.80×10^−5^). The *df* of a per-allele OR value is 2 in the additive genetic model analysis. ORs are computed using wild homozygous carriers of variant as the reference group in the dominance model analysis and non-risk homozygous carriers of variant as the reference group in the recessive model. The risk alleles are rs3737884, G and rs16850797, C, respectively.
(DOC)

Table S6 **Covariates analyses of risk genotypes in** ***ADIPOR1*** **associated with risk of T2D, CAD and T2D with CAD.** Variables were expressed as percentage, mean ± standard deviation, or median (interquartile range). Risk genotypes were defined on the basis of the genetic dominance model analysis in Table S5. Differences between covariates were compared using parametric (Student’s t-test for normally distributed variables) or nonparametric (Mann-Whitney U test for non-normally distributed variables) methods for continuous variables, and *P* values are obtained by Pearson’s χ^2^ for categorical variables. *Statistical significances are considered as *P*≤0.05; ** Statistical significances are considered as *P*≤0.017 according to Bonferroni correction under three independent hypotheses. CAD, coronary artery disease; T2D, type 2 diabetes; BMI, body mass index; SBP, systolic blood pressure; DBP, diastolic blood pressure; FBG, fasting plasma glucose; TG, triglycerides; TC, total cholesterol; HDL, high-density lipoprotein cholesterol; LDL, low-density lipoprotein cholesterol.
(DOC)
