# Supplementary figures and images for: Identification of Susceptibility Variants in ADIPOR1 Gene Associated with Type 2 Diabetes, Coronary Artery Disease and the Comorbidity of Type 2 Diabetes and Coronary Artery Disease
Source: PLoS One. 2014 Jun 26;9(6):e100339. doi: 10.1371/journal.pone.0100339 (PMC4072681; doi:10.1371/journal.pone.0100339)

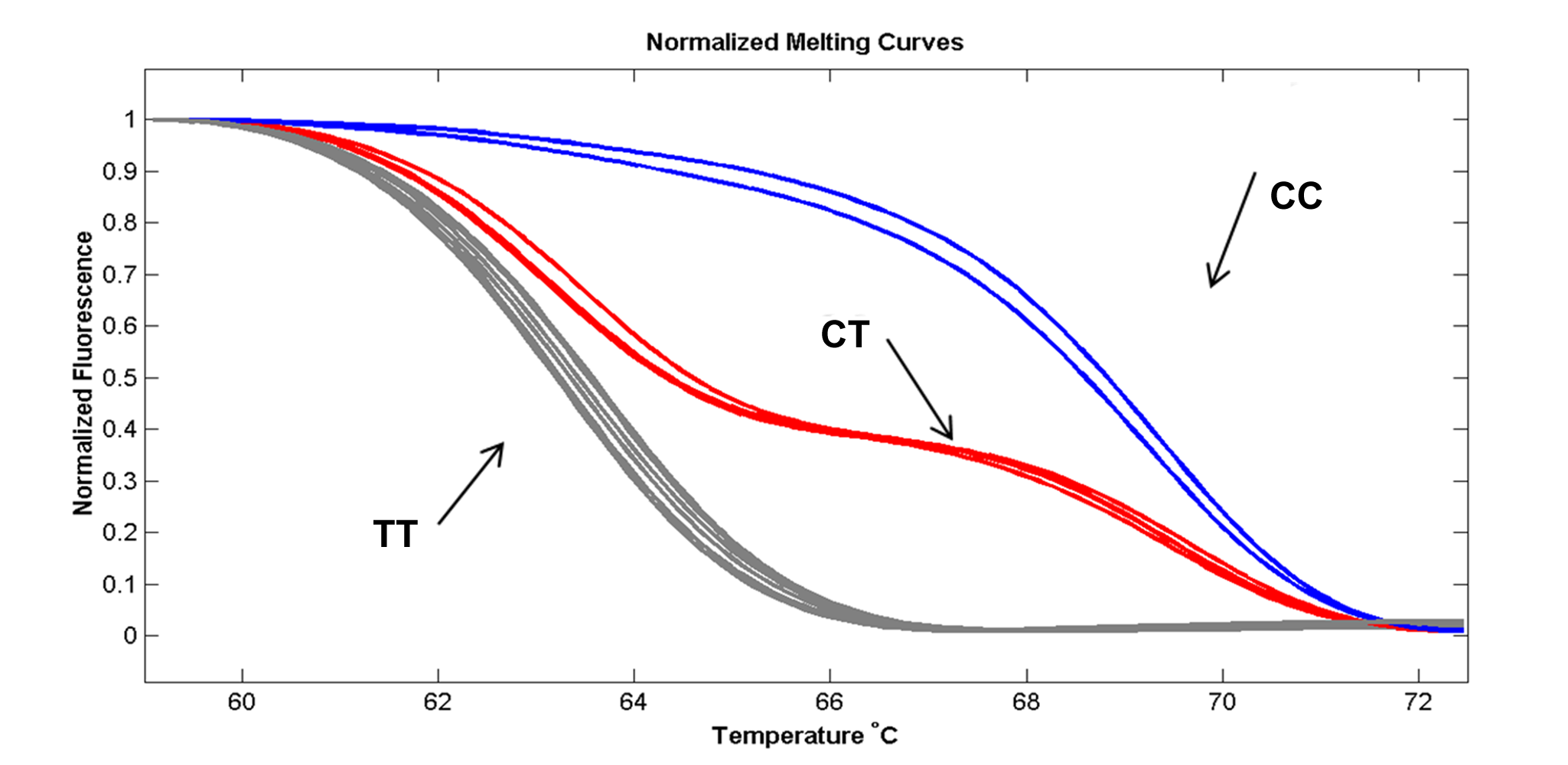

Supplement: Figure S2 — Genotyping results of rs12045862 using LightScanner TMHR-I 96. Melting curves results show homozygous wild genotype CC (blue curves), homozygous variant genotype TT (gray curves) and Heterozygous genotype CT (red curves). (TIF) [file pone.0100339.s002.tif]

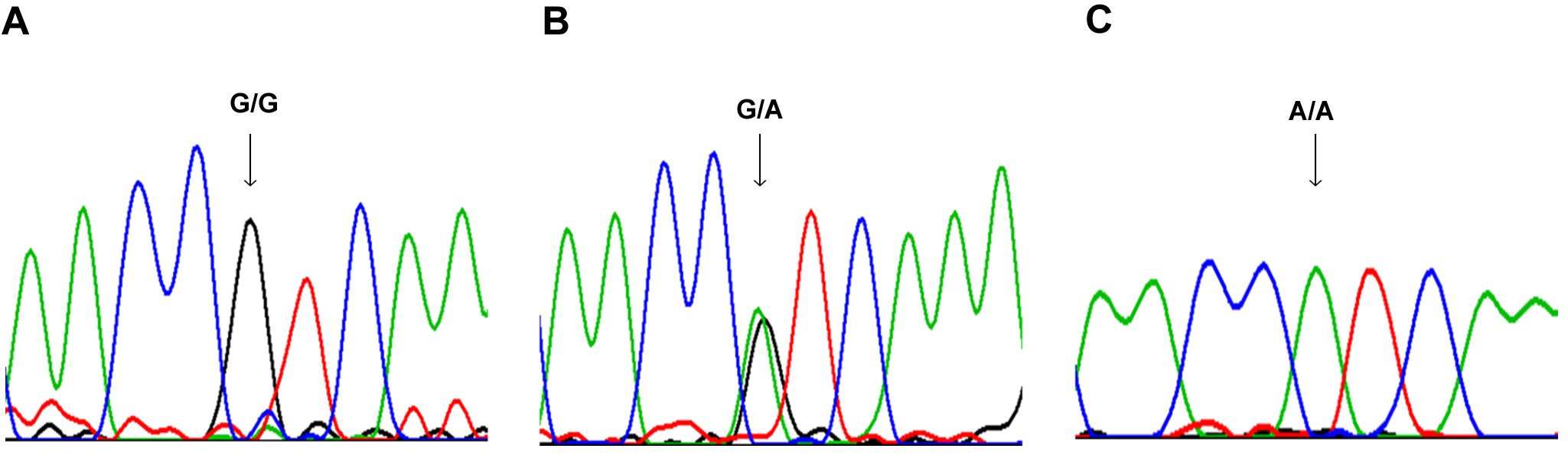

Supplement: Figure S3 — Sequencing results of rs1342387. The positions that the arrow pointed to are homozygous wild genotype GG (A), heterozygous genotype GA (B), and homozygous variant genotype AA (C), respectively. (TIF) [file pone.0100339.s003.tif]
