# Supplementary material for: Identification of Susceptibility Variants in ADIPOR1 Gene Associated with Type 2 Diabetes, Coronary Artery Disease and the Comorbidity of Type 2 Diabetes and Coronary Artery Disease
Source: PLoS One. 2014 Jun 26;9(6):e100339. doi: 10.1371/journal.pone.0100339 (PMC4072681; doi:10.1371/journal.pone.0100339)
Supplement: Table S1 — Basic information for genotyping. F: forward primer; R: reverse primer; P: probe; HRM: high-resolution melting; RFLP: restriction fragment length polymorphism. *rs12045862 was genotyped using unlabeled probe method of HRM. **R:A or G;Y:C or T;N:A,C,G or T. (DOC) [file pone.0100339.s004.doc]

**Table S1**. Basic information for genotyping reaction

| SNP | Allele | Genotype method | Amplicon length (bp) of PCR | Annealing temperature(℃) | Primer or probe sequences (5’- 3’) | restriction enzyme | cutting site(5’- 3’)^**^ | Fragment length (bp) after cutting |
| --- | --- | --- | --- | --- | --- | --- | --- | --- |
| rs7539542 | G>C | RFLP | 542 | 65 | F: GCACCCAGCCCTGAGAATCT | BsmAⅠ | GTCTC(N)↓ | 349/193 |
|  |  |  |  |  | R: CCGGCTAATCATGGAAGTGTGT |  |  |  |
| rs3737884 | C>T | RFLP | 305 | 56 | F: AGTAAGGGAAAGGGATAGAGT | PleⅠ | GAGTC(N)_4_↓ | 121/184 |
|  |  |  |  |  | R: TAATAGAGCCAGGGGACAAA |  |  |  |
| rs1342387 | T>C | RFLP | 758 | 58 | F: CCCGCTTCTAAGTCTCCAT | BccⅠ | CCATC(N)_4_↓ | 646/112 |
|  |  |  |  |  | R: TGAAGTACATATTTGGTCTGA |  |  |  |
| rs16850797 | G>C | RFLP | 264 | 61 | F: GGCTGAGGCAGGAGAAGTG | XagⅠ | CCTNN↓NNNAGG | 132/132 |
|  |  |  |  |  | R: GGGAGGGATGAGACATGAGATAG |  |  |  |
| rs12045862^*^ | C>T | HRM | 126 | 64 | F: TACAGATCCCTCCTTTGCCC | - | - | - |
|  |  |  |  |  | R: GCATGGATCCTAGATTAAGAACCC |  |  |  |
|  |  |  |  |  | P: TCATGTCTCATCCCTCCCCCAATTA |  |  |  |
| rs7514221 | C>T | RFLP | 291 | 58 | F: ACAACCTCAGGAACCGAAGT | HaeⅡ | RGCGC↓Y | 233/58 |
|  |  |  |  |  | R: GGAGAATGGGAAACTGACAA |  |  |  |

F:forward primer; R:reverse primer; P:probe; HRM: high-resolution melting; RFLP: restriction fragment length polymorphism.

^*^rs12045862 was genotyped using unlabeled probe method of HRM.

^**^R:A or G;Y:C or T;N:A,C,G or T.
