# Supplementary material for: Identification of Susceptibility Variants in ADIPOR1 Gene Associated with Type 2 Diabetes, Coronary Artery Disease and the Comorbidity of Type 2 Diabetes and Coronary Artery Disease
Source: PLoS One. 2014 Jun 26;9(6):e100339. doi: 10.1371/journal.pone.0100339 (PMC4072681; doi:10.1371/journal.pone.0100339)
Supplement: Table S2 — The multiple comparison results of clinical and demographic characteristics among groups. Variables were expressed as percentage, mean ± standard deviation, or median (interquartile range); Differences between characteristics were compared using parametric (Student’s t-test for normally distributed variables) or nonparametric (Mann-Whitney U test for non-normally distributed variables) methods for continuous variables, and Pearson’s χ2 analysis for categorical variables. CAD, coronary artery disease; T2D, type 2 diabetes; T2D+CAD: T2D with CAD; HDL, high-density lipoprotein cholesterol; LDL, low-density lipoprotein cholesterol; SBP, systolic blood pressure; DBP, diastolic blood pressure; FBG, fasting plasma glucose; TC, total cholesterol; TG, triglycerides; BMI, body mass index. Six independent hypotheses are tested with a significance level set at P≤0.008 (0.05/6) according to Bonferroni correction. (DOC) [file pone.0100339.s005.doc]

**Table S2**. The multiple comparison results of clinical and demographic characteristics among groups

| Characteristics | T2D+CAD vs.Control | T2D vs.Control | CAD vs.Control | T2D+CAD vs.T2D | T2D+CAD vs.CAD | T2D vs.CAD |
| --- | --- | --- | --- | --- | --- | --- |
| Age (years) | 0.286 | 0.037 | <0.001 | 0.148 | 0.001 | 0.141 |
| Male (%) | 0.057 | 0.009 | <0.001 | 0.440 | <0.001 | 0.001 |
| BMI(kg/m^2^) | <0.001 | <0.001 | <0.001 | <0.001 | <0.001 | 0.003 |
| SBP (mmHg) | <0.001 | <0.001 | <0.001 | <0.001 | <0.001 | 0.007 |
| DBP(mmHg) | <0.001 | 0.001 | <0.001 | <0.001 | 0.001 | 0.022 |
| FBG(mmol/l) | <0.001 | <0.001 | 0.002 | <0.001 | <0.001 | <0.001 |
| TG (mmol/l) | <0.001 | 0.01 | <0.001 | <0.001 | 0.358 | 0.001 |
| TC (mmol/l) | <0.001 | 0.059 | <0.001 | <0.001 | 0.668 | <0.001 |
| HDL-C (mmol/l) | <0.001 | <0.001 | <0.001 | <0.001 | 0.509 | <0.001 |
| LDL-C (mmol/l) | 0.098 | 0.236 | 0.636 | 0.067 | 0.326 | 0.147 |

Variables were expressed as percentage, mean±standard deviation, or median (interquartile range); Differences between characteristics were compared using parametric (Student’s *t*-test for normally distributed variables) or nonparametric (Mann-Whitney U test for non-normally distributed variables) methods for continuous variables, and Pearson's χ^２^ analysis for categorical variables. CAD, coronary artery disease;T2D, type 2 diabetes; T2D+CAD,T2D with CAD; HDL, high-density lipoprotein cholesterol; LDL, low-density lipoprotein cholesterol; SBP, systolic blood pressure; DBP, diastolic blood pressure; FBG, fasting plasma glucose; TC, total cholesterol; TG, triglycerides; BMI, body mass index. Six independent [hypotheses](http://en.wikipedia.org/wiki/Statistical_hypothesis_testing) are tested with a significance level set at *P*<0.008 (0.05/6) according to Bonferroni correction.
