# Supplementary material for: Identification of Susceptibility Variants in ADIPOR1 Gene Associated with Type 2 Diabetes, Coronary Artery Disease and the Comorbidity of Type 2 Diabetes and Coronary Artery Disease
Source: PLoS One. 2014 Jun 26;9(6):e100339. doi: 10.1371/journal.pone.0100339 (PMC4072681; doi:10.1371/journal.pone.0100339)
Supplement: Table S3 — Allelic distribution of the 6 SNPs in ADIPOR1 in our study. SNPs: single nucleotide polymorphism; CAD, coronary artery disease; T2D, type 2 diabetes; T2D+CAD: T2D with CAD. *These alleles were defined on the basis of the alleles contrast in this study. **The former is ancestral allele. *** UTR-3: untranslated region. (DOC) [file pone.0100339.s006.doc]

**Table S3**. Allelic distribution of the 6 SNPs in *ADIPOR1* in our study.

| SNPs | Function | Alternative alleles ^**^ | Minor allele^*^ | Minor allele frequencies | | | |
| --- | --- | --- | --- | --- | --- | --- | --- |
|  |  |  |  | T2D+CAD | CAD | T2D | Control |
| rs7539542 | UTR-3^***^ | G,C | C | 0.34 | 0.32 | 0.39 | 0.38 |
| rs3737884 | Intron | A,G | A | 0.20 | 0.19 | 0.22 | 0.36 |
| rs1342387 | Intron | G,A | A | 0.37 | 0.37 | 0.38 | 0.36 |
| rs16850797 | Intron | G,C | C | 0.37 | 0.26 | 0.33 | 0.24 |
| rs12045862 | Intron | C,T | C | 0.37 | 0.39 | 0.32 | 0.40 |
| rs7514221 | Intron | T,C | C | 0.14 | 0.16 | 0.15 | 0.11 |

SNPs: single nucleotide polymorphism; CAD, coronary artery disease;T2D, type 2 diabetes ; T2D+CAD:T2D with CAD

^*^These alleles were defined on the basis of the alleles contrast in this study.

^**^The former was ancestral allele.

^***^ UTR-3: untranslated region.
