# Supplementary material for: Identification of Susceptibility Variants in ADIPOR1 Gene Associated with Type 2 Diabetes, Coronary Artery Disease and the Comorbidity of Type 2 Diabetes and Coronary Artery Disease
Source: PLoS One. 2014 Jun 26;9(6):e100339. doi: 10.1371/journal.pone.0100339 (PMC4072681; doi:10.1371/journal.pone.0100339)
Supplement: Table S4 — Allelic distribution of SNPs in our study, HapMap and Ensembl database. HapMap database is from International HapMap Project (http://hapmap.ncbi.nlm.nih.gov/). Ensembl database is from the 1000 Genomes data (http://asia.ensembl.org/index.html). HCB or CHB: Han Chinese in Beijing, China. CHS: Southern Han Chinese. JPT: Japanese in Tokyo, Japan. CEU: Utah residents with Northern and Western European ancestry from the CEPH collection. YRI: Yoruba in Ibadan, Nigeria (Sub-Saharan African). Major: major allele frequency. Minor: minor allele frequency. HWE: Hardy-Weinberg equilibrium. The character “−” represents “unavailable”. *Numbers of sample size. **P values are derived from comparing the Control group with other groups, respectively. Statistical significances are considered as P≤0.013 (0.05/4, compared Control with HapMap database) or 0.01 (0.05/5, compared Control with Ensembl database). P values are obtained by Pearson’s χ2 analysis. (DOC) [file pone.0100339.s007.doc]

**Table S4**. Allelic distribution of SNPs in our study, HapMap and Ensembl databases.

|  | This study | HapMap | | | | Ensembl | | | | |
| --- | --- | --- | --- | --- | --- | --- | --- | --- | --- | --- |
| rs7539542 | Control | HCB | JPT | CEU | YRI | HCB | CHS | JPT | CEU | YRI |
| Numbers^*^ | 145 | 42 | 43 | 59 | 59 | 97 | 100 | 89 | 85 | 88 |
| Major | G=0.62 | G=0.69 | G=0.81 | C=0.71 | G=0.86 | G=0.63 | G=0.61 | G=0.78 | C=0.69 | G=0.73 |
| Minor | C=0.38 | C=0.31 | C=0.19 | G=0.29 | C=0.14 | C=0.37 | C=0.39 | C=0.22 | G=0.31 | C=0.27 |
| *P*_HWE_ | 0.09 | 0.53 | 0.66 | 1.00 | 0.93 | 0.25 | 0.45 | 0.43 | 0.98 | 0.69 |
| *P*^**^ | - | 0.22 | 7.05×10^-4^ | 1.60×10^-9^ | 1.01×10^-6^ | 0.8 | 0.87 | 2.30×10^-4^ | 1.14×10^-10^ | 0.01 |
|  | This study | HapMap | | | | Ensembl | | | | |
| rs3737884 | Control | HCB | JPT | CEU | YRI | HCB | CHS | JPT | CEU | YRI |
| Numbers | 145 | 45 | 45 | 60 | 60 | 97 | 100 | 89 | 85 | 88 |
| Major | G=0.63 | G=0.80 | G=0.92 | G=1.00 | G=0.70 | G=0.78 | G=0.78 | G=0.90 | G=1.00 | G=0.72 |
| Minor | A=0.36 | A=0.20 | A=0.08 | A=0.00 | C=0.30 | A=0.22 | A=0.22 | A=0.10 | A=0.00 | A=0.28 |
| *P*_HWE_ | 0.47 | 1.00 | 1.00 | "- | 1.00 | 0.14 | 0.34 | 0.92 | - | 0.56 |
| *P*^**^ | - | 4.10×10^-3^ | 2.37×10^-7^ | 8.88×10^-15^ | 0.23 | 6.43×10^-4^ | 4.92×10^-4^ | 4.78×10^-10^ | 8.90×10^-15^ | 0.08 |
|  | This study | HapMap | | | | Ensembl | | | | |
| rs16850797 | Control | HCB | JPT | CEU | YRI | HCB | CHS | JPT | CEU | YRI |
| Numbers | 145 | 45 | 45 | 59 | 60 | 97 | 100 | 89 | 85 | 88 |
| Major | G=0.76 | G=0.77 | G=0.69 | G=1.00 | G=0.99 | G=0.76 | G=0.77 | G=0.67 | G=0.99 | G=1.00 |
| Minor | C=0.24 | C=0.23 | C=0.31 | C=0.00 | C=0.01 | C=0.24 | C=0.23 | C=0.33 | C=0.01 | C=0.00 |
| *P*_HWE_ | 0.80 | 0.75 | 0.37 | - | 1.00 | 0.39 | 0.87 | 0.83 | 0.91 | - |
| *P*^**^ | - | 0.88 | 0.19 | 3.96×10^-12^ | 1.39×10^-8^ | 0.91 | 0.77 | 0.05 | 2.53×10^-13^ | 5.41×10^-15^ |
|  | This study | HapMap | | | | Ensembl | | | | |
| rs7514221 | Control | HCB | JPT | CEU | YRI | HCB | CHS | JPT | CEU | YRI |
| Numbers | 145 | 41 | 86 | 113 | 112 | 97 | 100 | 85 | 85 | 88 |
| Major | T=0.89 | T=0.87 | T=0.87 | T=0.57 | T=0.53 | T=0.845 | T=0.86 | T=0.85 | T=0.57 | T=0.54 |
| Minor | C=0.11 | C=0.13 | C=0.13 | C=0.43 | C=0.47 | C=0.155 | C=0.14 | C=0.15 | C=0.43 | C=0.46 |
| *P*_HWE_ | 0.77 | 0.75 | 0.66 | 0.20 | 0.15 | 0.80 | 0.09 | 0.39 | 0.56 | 0.05 |
| *P*^**^ | - | 0.49 | 0.49 | 1.83×10^-17^ | 1.23×10^-20^ | 0.12 | 0.21 | 0.15 | 1.44×10^-15^ | 4.97×10^-18^ |

HapMap database is from International HapMap Project (http://hapmap.ncbi.nlm.nih.gov/). Ensembl database is from the 1000 Genomes data (http://asia.ensembl.org/index.html).HCB or CHB: Han Chinese in Beijing, China. CHS: Southern Han Chinese. JPT: Japanese in Tokyo, Japan. CEU: Utah residents with Northern and Western European ancestry from the CEPH collection. YRI: Yoruba in Ibadan, Nigeria (Sub-Saharan African). Major: major allele frequency. Minor: minor allele frequency. HWE: Hardy-Weinberg equilibrium. The character “-” represents “unavailable”. ^*^Numbers of sample size. ^**^*P* values are derived from comparing the control group with other groups respectively. Statistical significances are considered as *P*<0.013 (0.05/4,compared Control with HapMap database) or 0.01 (0.05/5,compared Control with Ensembl database). *P* values are obtained by Pearson's χ^２^ analysis.
