# Supplementary material for: Identification of Susceptibility Variants in ADIPOR1 Gene Associated with Type 2 Diabetes, Coronary Artery Disease and the Comorbidity of Type 2 Diabetes and Coronary Artery Disease
Source: PLoS One. 2014 Jun 26;9(6):e100339. doi: 10.1371/journal.pone.0100339 (PMC4072681; doi:10.1371/journal.pone.0100339)
Supplement: Table S5 — Association of rs3737884 and rs16850797 with diseases in three types of genetic models. CAD, coronary artery disease; T2D, type 2 diabetes; T2D+CAD: T2D with CAD; OR, odds ratios; CI, confidence interval. All OR and P values are obtained by Pearson’s χ2 or unconditional logistic regression and adjusted for gender, age and body mass index. All variants with nominal significance (P≤0.05) are listed; the threshold for significance by Bonferroni correction is 0.05/3 = 0.017 (three independent hypotheses: T2D vs. Control, CAD vs. Control, T2D with CAD vs. Control).*P value that can pass multiple testing correction (P≤0.017); E indicates the power of the base-10 exponent (i.e. 9.80E-05 = 9.80×10^−5^). The df of a per-allele OR value is 2 in the additive genetic model analysis. ORs are computed using wild homozygous carriers of variant as the reference group in the dominance model analysis and non-risk homozygous carriers of variant as the reference group in the recessive model. The risk alleles are rs3737884, G and rs16850797, C, respectively. (DOC) [file pone.0100339.s008.doc]

Table S5. Association of rs3737884 and rs16850797with diseases in three types of genetic models

| Genetic models | T2D | Control | OR^a^ (95% CI) | *P*^a^ | CAD | Control | OR^b^ (95% CI) | *P*^b^ | T2D+CAD | Control | O^Rc^(95% CI) | *P*^c^ |
| --- | --- | --- | --- | --- | --- | --- | --- | --- | --- | --- | --- | --- |
| **rs3737884** | | | | | | | | | | | | |
| additive model | | | | | | | | | | | | |
| GG vs. GA vs. AA | 98/62/5 | 61/63/21 | 1.96(1.33-2.89) | 6.30E-04^*^ | 114/53/6 | 61/63/21 | 2.42(1.55-3.77) | 9.80E-05^*^ | 111/57/6 | 61/63/21 | 2.42(1.51-3.89) | 2.48E-04^*^ |
| dominance model | | | | | | | | | | | | |
| GG+GA | 160 | 124 | 1.89(1.12-3.20) | 0.017^*^ | 167 | 124 | 5.11(1.61-16.20) | 5.64E-03^*^ | 168 | 124 | 8.63(2.50-29.76) | 6.45E-04^*^ |
| AA | 5 | 21 | 1.00(reference) |  | 6 | 21 | 1.00(reference) |  | 6 | 21 | 1.00(reference) |  |
| recessive model | | | | | | | | | | | | |
| GG | 98 | 61 | 1.95(1.21-3.14) | 0.006^*^ | 114 | 61 | 2.62(1.52-4.50) | 4.88E-04^*^ | 111 | 61 | 2.30(1.28-4.11) | 0.005^*^ |
| GA+AA | 67 | 84 | 1.00(reference) |  | 59 | 84 | 1.00(reference) |  | 63 | 84 | 1.00(reference) |  |
| **rs16850797** | | | | | | | | | | | | |
| additive model | | | | | | | | | | | | |
| CC vs. GC vs. GG | 8/89/63 | 9/52/84 | 1.77（1.169-2.67） | 0.007^*^ | 13/64/96 | 9/52/84 | 1.21(0.79-1.87) | 0.380 | 28/73/73 | 9/52/84 | 1.71(1.11-2.62) | 0.014^*^ |
| dominance model | | | | | | | | | | | | |
| CC+GC | 97 | 61 | 2.43（1.49-3.97） | 3.76E-04^*^ | 77 | 61 | 1.24(0.73-2.12) | 0.420 | 101 | 61 | 1.78(1.00-3.16) | 0.050 |
| GG | 63 | 84 | 1.00(reference) |  | 96 | 84 | 1.00(reference) |  | 73 | 84 | 1.00(reference) |  |
| recessive model | | | | | | | | | | | | |
| CC | 8 | 9 | 0.68(0.24-1.91) | 0.464 | 13 | 9 | 1.37(0.46-4.13) | 0.570 | 28 | 9 | 2.89(1.11-7.50) | 0.030 |
| GC+GG | 152 | 136 | 1.00(reference) |  | 160 | 136 | 1.00(reference) |  | 146 | 136 | 1.00(reference) |  |

CAD, coronary artery disease; T2D, type 2 diabetes ; T2D+CAD:T2D with CAD; OR, odds ratios; CI, confidence interval.

All OR and *P* values are obtained by Pearson's χ^２^ or unconditional logistic regression and adjusted for gender, age and body mass index. All variants with nominal signiﬁcance (*P*<0.05) are listed; the threshold for signiﬁcance by Bonferroni correction is 0.05/3=0.017 (three independent [hypotheses](http://en.wikipedia.org/wiki/Statistical_hypothesis_testing): T2D vs. Control, CAD vs.Control,T2D with CAD vs. Control).^*^*P* value that can pass multiple testing correction (*P*<0.017) ;E indicates the power of the base-10 exponent (i.e. 9.80E-05= 9.80×10^-5^). The *df* of a per-allele OR value is 2 in the additive genetic model analysis. ORs are computed using wild homozygous carriers of variant as the reference group in the dominance model analysis and non-risk homozygous carriers of variant as the reference group in the recessive model. The risk alleles are rs3737884, G and rs16850797,C, respectively.
