# Supplementary material for: Identification of Susceptibility Variants in ADIPOR1 Gene Associated with Type 2 Diabetes, Coronary Artery Disease and the Comorbidity of Type 2 Diabetes and Coronary Artery Disease
Source: PLoS One. 2014 Jun 26;9(6):e100339. doi: 10.1371/journal.pone.0100339 (PMC4072681; doi:10.1371/journal.pone.0100339)
Supplement: Table S6 — Covariates analyses of risk genotypes in ADIPOR1 associated with risk of T2D, CAD and T2D with CAD. Variables were expressed as percentage, mean ± standard deviation, or median (interquartile range). Risk genotypes were defined on the basis of the genetic dominance model analysis in Table S5. Differences between covariates were compared using parametric (Student’s t-test for normally distributed variables) or nonparametric (Mann-Whitney U test for non-normally distributed variables) methods for continuous variables, and P values are obtained by Pearson’s χ2 for categorical variables. *Statistical significances are considered as P≤0.05; ** Statistical significances are considered as P≤0.017 according to Bonferroni correction under three independent hypotheses. CAD, coronary artery disease; T2D, type 2 diabetes; BMI, body mass index; SBP, systolic blood pressure; DBP, diastolic blood pressure; FBG, fasting plasma glucose; TG, triglycerides; TC, total cholesterol; HDL, high-density lipoprotein cholesterol; LDL, low-density lipoprotein cholesterol. (DOC) [file pone.0100339.s009.doc]

**Table S6.**  Clinical covariates analyses of risk genotypes in *ADIPOR1* associated with risk of T2D, CAD and T2D with CAD.

|  | rs16850797(CC+CG) | | | | rs3737884(GG+GA) | | | |
| --- | --- | --- | --- | --- | --- | --- | --- | --- |
| Covariates | T2D+CAD | Control | Z or t or χ^2^ | *P^*^*-value | T2D+CAD | Control | Z or t or χ^2^ | *P^**^*-value |
| Age | 61(55.3-67.0) | 65（52.5-70.0） | -1.43 | 0.15 | 61.27±11.65 | 62.31±11.52 | 0.77 | 0.44 |
| Male, n (%) | 69(68.3) | 34(55.7) | 2.60 | 0.11 | 117(69.6) | 76(61.3) | 0.22 | 0.14 |
| BMI | 26.56±3.13 | 22.45±2.87 | -8.23 | 7.75×10^-14^ | 25.56(23.85-27.97) | 22(20.81-24.23) | -8.67 | 4.33×10^-18^ |
| SBP | 133.0(120.0-150.0) | 110.0（102.5-120.0） | -7.75 | 9.19×10^-15^ | 130(120-150) | 110(104.5-120) | -10.39 | 2.81×10^-25^ |
| DBP | 80(70-90) | 70(60-70) | -6.23 | 4.63×10^-10^ | 80(70-90) | 70(60-76) | -7.28 | 3.4×10^-13^ |
| FBG | 7.00(5.52-9.20) | 5.10（4.85-5.30) | -7.80 | 6.08×10^-15^ | 7.10(5.8-9.0) | 5.1(4.9-5.3) | -10.76 | 5.16×10^-27^ |
| TG | 1.66(1.27-2.33) | 1.03(0.84-1.31) | -6.09 | 1.14×10^-9^ | 1.65(1.08-2.31) | 1.04(0.83-1.35) | -7.40 | 1.35×10^-13^ |
| LDL-C | 2.94±1.01 | 2.90±0.83 | -0.26 | 0.79 | 2.78±0.95 | 2.84±0.80 | 0.60 | 0.55 |
| HDL-C | 0.96±0.23 | 1.63±0.30 | 15.50 | 4.94×10^-33^ | 0.92(0.79-1.11) | 1.57(1.39-1.85) | -12.84 | 9.78×10^-38^ |
| TC | 4.44±1.11 | 5.15±0.96 | 4.08 | 7.33×10^-5^ | 4.30±1.17 | 5.05±0.98 | 5.69 | 3.25×10^-8^ |
|  | rs16850797(CC+CG) | | | | rs3737884(GG+GA) | | | |
| Covariates | T2D | Control | Z or t or χ^2^ | *P^*^*-value | T2D | Control | Z or t or χ^2^ | *P^**^*-value |
| Age | 60.27±11.81 | 61.48±12.76 | 0.42 | 0.67 | 59.34±12.18 | 62.31±11.52 | 2.09 | 0.04 |
| Male, n (%) | 33(68.8) | 15(51.7) | 2.23 | 0.14 | 117(73.1) | 76(61.3) | 4.49 | 0.03 |
| BMI | 23.16(21.53-26.37) | 21.44(21.00-22.89) | -2.39 | 0.02 | 23.51(21.87-26.12) | 22(20.81-24.23) | -4.32 | 1.53×10^-5^ |
| SBP | 121.25±13.03 | 111.48±9.81 | -3.48 | 8.38×10^-4^ | 120(110-126.8) | 110(104.5-120) | -5.01 | 5.38×10^-7^ |
| DBP | 72.14±10.26 | 67.77±7.80 | -2.85 | 4.95×10^-3^ | 70(70-80) | 70(60-76) | -3.25 | 0.001 |
| FBG | 6.82(6.29-7.49) | 5.20(4.90-5.35) | -7.07 | 1.57×10^-12^ | 6.95(6.30-7.85) | 5.1(4.9-5.3) | -13.59 | 4.81×10^-42^ |
| TG | 1.11(0.82-1.64) | 0.96(0.85-0.96) | -1.39 | 0.17 | 1.21(0.95-1.64) | 1.04(0.83-1.35) | -3.38 | 7.21×10^-4^ |
| LDL-C | 2.88±0.92 | 2.91±0.73 | 0.11 | 0.91 | 2.95(2.40-3.56) | 2.84±0.80 | -1.39 | 0.17 |
| HDL-C | 1.26±0.34 | 1.66±0.27 | 5.48 | 5.43×10^-7^ | 1.26(1.04-1.48) | 1.61±0.31 | 0.47 | 1.42×10^-15^ |
| TC | 4.72±1.07 | 5.16±0.92 | 1.86 | 0.07 | 4.89±1.09 | 5.05±0.98 | 0.17 | 0.19 |

**Table S6**. continue

|  | rs3737884(GG+GA) | | | |
| --- | --- | --- | --- | --- |
| Covariates | CAD | Control | Z or t or χ^2^ | *P^**^*-value |
| Age | 58.13±10.64 | 62.31±11.52 | 3.20 | 0.002 |
| Male, n (%) | 146(87.4) | 76(61.3) | 26.87 | 2.18×10^-7^ |
| BMI | 24.88(22.50-26.64) | 22(20.81-24.23) | -6.09 | 1.11×10^-9^ |
| SBP | 120(120-130) | 110(104.5-120) | -7.61 | 2.78×10^-14^ |
| DBP | 77(70-80) | 70(60-76) | -5.30 | 1.19×10^-7^ |
| FBG | 5.30(4.89-5.76) | 5.1(4.9-5.3) | -3.09 | 0.002 |
| TG | 1.48(1.06-2.22) | 1.04(0.83-1.35) | -6.53 | 6.38×10^-11^ |
| LDL-C | 2.73(2.12-3.52) | 2.67(2.30-3.29) | 0.11 | 0.91 |
| HDL-C | 0.94(0.81-1.21) | 1.57(1.39-1.85) | -13.05 | 6.65×10^-39^ |
| TC | 4.33±1.12 | 5.05±0.98 | 5.7 | 2.97×10^-8^ |

Variables were expressed as percentage, mean ± standard deviation, or median (interquartile range). Risk genotypes were defined on the basis of the genetic dominance model analysis in Table S5. Differences between covariates were compared using parametric (Student’s t-test for normally distributed variables) or nonparametric (Mann-Whitney U test for non-normally distributed variables) methods for continuous variables, and *P* values are obtained by Pearson's χ^２^ for categorical variables. ^*^Statistical significances are considered as *P*<0.05; ^**^ Statistical significances are considered as *P*<0.017 according to Bonferroni correction under three independent [hypotheses](http://en.wikipedia.org/wiki/Statistical_hypothesis_testing).

CAD, coronary artery disease;T2D,type 2 diabetes ; BMI, body mass index; SBP, systolic blood pressure; DBP, diastolic blood pressure; FBG, fasting plasma glucose; TG, triglycerides; TC, total cholesterol; HDL, high-density lipoprotein cholesterol; LDL, low-density lipoprotein cholesterol.
